# Awareness and perceptions of virtual reality in physiotherapy: a cross-sectional study among students and clinicians in Palestine

**DOI:** 10.1186/s12909-025-08117-3

**Published:** 2025-10-30

**Authors:** Amer Ghrouz, Fahed Herbawi, Duaa Abualkhair, Rami Jaber, Haneen Dhaidel, Hala Salah, Amani Anabtawi, Azza Alsharif, Malik Alqub, Mustafa Ghanim, Hazem Abu Nijmeh

**Affiliations:** 1 Department of Applied and Allied Medical Sciences, Faculty of Medicine and Allied Medical Sciences, An-Najah National University, Nablus, Palestine; 2 Department of Biomedical Sciences and Basic Clinical Skills, Faculty of Medicine and Allied Medical Sciences, An-Najah National University, P.O. Box:7, Nablus, Palestine

**Keywords:** Virtual reality, Augmented reality, Rehabilitation, Physiotherapy, Education

## Abstract

**Background:**

Virtual reality(VR) and augmented reality(AR) are increasingly used in physiotherapy to improve patient engagement and recovery outcomes. However, their adoption in Palestine remains limited. Understanding students’ and clinicians’ awareness and impressions is critical for planning future VR incorporation into physiotherapy education and practice.

**Objectives:**

To explore and compare the awareness and perceptions of VR/AR technologies in physiotherapy among final-year physiotherapy students and practicing clinicians in Palestine.

**Methods:**

This cross-sectional study was carried out from December 2024 to May 2025 involved 500 participants recruited via convenience sampling from accredited universities and rehabilitation centers throughout Palestine. A well-structured, self-administered questionnaire was developed to assess participants’ knowledge, clinical familiarity, training needs, and future perspectives regarding VR in physiotherapy among final-year students and clinicians. A pilot studywereth 30 participants was conducted to determine the questionnaire’s content validity and reliability, demonstrating a good internal consistency with a Cronbach’s alpha of 0.80.

**Results:**

Most participants were female (70.6%) and aged 18–30 years (93.6%), with 70% final-year physiotherapy students and 30% clinicians. Clinicians reported higher VR/AR awareness (62.3% vs. 52.6%, *p* = 0.017) and greater participation in related workshops (*p* < 0.05). Both groups identified motion tracking as defining rehabilitation technology. High cost was the most cited disadvantage, while neurological and musculoskeletal applications were viewed as most suitable. Although 51.2% were neutral on ethical concerns, over half believed current training was inadequate, and most preferred combining VR with traditional methods (89%). Overall, 91.8% supported specialized educational programs to enhance clinical application, engagement, and progress tracking.

**Conclusion:**

The findings demonstrate moderate VR/AR awareness among Palestinian physiotherapists, but their limited hands-on experience and institutional support hinder widespread adoption. Clinicians are more familiar than students, indicating the need for earlier curricular exposure.

## Introduction

Virtual reality (VR) is widely recognized as a revolutionary technology within current healthcare systems, that significantly enhances patient care delivery and therapeutic engagement methods. Its applications span throughout various medical domains, including surgical models, pain alterations, and psychological effects. Specifically, in physiotherapy VR technologies have significant received attention because of their capacity to create interactive and immersive environments compared with the conventional physical therapy practices [1, 2].

A recent study has been demonstrated that VR is used in various physiotherapy approaches and that VR is a promising tool for rehabilitation [1, 3]. In rehabilitation, VR provides real-time biomechanical feedback, enhances patient motivation, and enables the simulation of functional tasks and activities, thereby benefiting both patients and especially in patient adherence, and for the physical therapists. These characteristics are especially helpful when treating neurological and musculoskeletal conditions. and demonstrated positive gains in range of motion, gait, pain management, balance, and motor function [2, 4]. For example, VR-based interventions for shoulder rehabilitation have demonstrated remarkable improvements in patient engagement and satisfaction [2, 4].

Nevertheless, the use of VR technology in physiotherapy assessment and rehabilitation is delayed by systemic barriers, such as insufficient awareness, training, and infrastructural support. Many investigations suggest that while physiotherapists and students recognize the theoretical importance of new health technologies such as VR, their daily practical application with patients remains [5]. Furthermore, clinicians frequently mentioned inadequate preparedness to combine VR into treatment regimens, citing deficits in educational resources and institutional certification [1, 6].

Although the awareness level and use of therapeutic VR among physiotherapists has been reported for some countries such as Germany, a survey by Elser et al. (2025) of 296 physiotherapists revealed that only 2.7% had used VR in 2024 and that 67.2% had never heard of therapeutic VR. Despite this, many physiotherapists expressed openness to future use [7]. However, there is no existing literature or research regarding the resources, usefulness, or knowledge of clinical use in Palestine or other Middle East countries.

To the best of our knowledge, no research has examined physical therapists’ awareness of the benefits of virtual reality. Thus, the purpose of this study was to determine how well-informed physical therapists are about the use of virtual reality technology in patient evaluation and recovery. We hypothesized that the majority of physiotherapists in Palestine are not well-versed in the mentioned technology and how it is used.

## Methodology

### Study design and setting

A cross-sectional survey was conducted to assess the awareness and perceptions of virtual reality (VR) applications in physiotherapy among two target populations in Palestine: final-year physiotherapy students and practicing physiotherapists (PTs). Participants were recruited from multiple accredited universities and physiotherapy and rehabilitation centers across different regions of Palestine between December 2024 and May 2025.

### Participants

The study included two primary groups: (1) final-year physiotherapy students enrolled in accredited Palestinian universities, and (2) licensed physiotherapists actively engaged in clinical practice. Final-year (4th-year) students were specifically included because they represent individuals closest to professional entry, with substantial theoretical and clinical exposure, making their perspectives comparable to practicing clinicians.

Eligibility criteria required participants to be either final-year students or clinicians currently involved in direct patient care. Exclusion criteria were applied to individuals unable to provide informed consent, those with insufficient proficiency in Arabic (the language of the survey), and those with significant cognitive or communication difficulties that prevented independent completion of the questionnaire. Cognitive eligibility was judged informally by participants’ ability to understand the consent form and complete the pilot questionnaire, rather than through a standardized screening tool.

### Sample size

The required sample size was estimated using Cochran’s formula for cross-sectional surveys:

$$\mathrm n=(\mathrm Z^2\;\times\;\mathrm p\;\times\;(1\;-\;\mathrm p))\;/\;\mathrm e^2$$

Assuming a 95% confidence level (Z = 1.96), an expected prevalence of 50% (*p* = 0.5; due to lack of prior regional data), and a margin of error of 5% (e = 0.05), the minimum sample size was calculated as 385. To accommodate potential non-response, 15% was added, increasing the target to 443 participants. Ultimately, 500 individuals completed the questionnaire, exceeding the required sample size and strengthening the statistical power for subgroup analyses.

### Sampling method and recruitment

Due to logistical and resource constraints, convenience sampling was used. While this non-probabilistic approach may introduce selection bias, it facilitated the inclusion of a large number of participants across different regions within a limited timeframe. Recruitment was conducted through online dissemination of the questionnaire via official channels of universities and rehabilitation centers. Participation was voluntary, and all participants provided informed consent prior to enrollment.

### Instrument development and validation

Data were collected using a structured, self-administered questionnaire developed following a comprehensive review of literature on VR/AR applications in physiotherapy and rehabilitation. The questionnaire comprised four sections:

1. Awareness and knowledge of VR/AR in physiotherapy and rehabilitation (5 items).
2. Familiarity with VR/AR applications in clinical practice (5 items).
3. Training needs and future directions for VR/AR in physiotherapy (6 items).
4. Perspectives on the future of VR/AR technologies in the field (4 items).

Each item was measured on either a dichotomous (Yes/No), categorical, or 5-point Likert scale, depending on the construct. Scores for each domain were calculated by summing responses, with higher scores indicating greater awareness, familiarity, or positive perceptions. The minimum and maximum possible scores for each domain were specified, ensuring transparency for future replication.

### Content validity

Content validity was assessed by a panel of five experts: three senior physiotherapy faculty members and two clinicians with over ten years of experience in rehabilitation practice. The experts evaluated the questionnaire for clarity, relevance, comprehensiveness, and cultural appropriateness. Feedback was incorporated to refine the wording and ensure the questionnaire adequately captured the intended domains.

### Pilot testing and reliability

A pilot study was conducted with 30 participants (15 students and 15 clinicians) who met the inclusion criteria. Feedback confirmed the clarity of the questions, and only minor wording changes were required. A preliminary psychometric analysis demonstrated acceptable internal consistency, with a Cronbach’s alpha of 0.80 across all questionnaire domains.

### Data analysis

Data were analyzed using SPSS version 23.0. Descriptive statistics (frequencies and percentages) summarized participant characteristics and responses. Chi-square tests were used to compare categorical variables between students and clinicians, while Mann–Whitney U tests were applied for non-normally distributed continuous data. A p-value ≤ 0.05 was considered statistically significant.

### Ethical considerations

The study received approval from the Institutional Review Board (IRB) of An-Najah National University, Nablus, Palestine (Approval No: AAMS.Dec.2024/24). Written informed consent was obtained from all participants. Confidentiality and anonymity were maintained, and the study adhered to the principles of the Declaration of Helsinki.

## Results

### Socio‑demographic characteristics

As shown in Table 1, the majority of participants were female (70.6%), and most were aged between 18 and 30 years (93.6%). Regarding occupation status, 70% were final-year students of physiotherapy bachelor’s degree and 30% were practicing physiotherapists (Practicing PTs). In terms of work experience, most participants (93%) had 0–5 years of experience, while smaller proportions had 6–10 years (2%), 11–16 years (0.6%), and more than 16 years of experience (4.4%). Regarding working hours per week, 72.8% worked 21–30 h, with the remainder working less than 10 h (11.2%), 11–20 h (10%), or more than 30 h (6%). Concerning academic qualifications, the majority (87.8%) held a bachelor’s degree or were in their fourth year of study, whereas the remaining participants had a diploma (7.2%), a master’s degree (4.2%), or a doctorate degree (0.8%).

**Table 1:** Distribution of participants based on their socio-demographic characteristics

		Participants (*n*=500)	Percentages %
Gender	Male	147	29.4%
	Female	353	70.6%
Age	18–30	468	93.6%
	31–40	12	2.4%
	41–50	16	3.2%
	50 and above	4	0.8%
Groups	Final- year students	350	70%
	Practicing PTs	150	30%
Years of Experience	Internship experience	115	23%
	0-5years	350	70%
	6-10years	10	2%
	11-16years	3	0.6%
	16 years and above	22	4.4%
Hours of work per week	Less than 10 hours	56	11.2%
	11–20 hours	50	10%
	21–30 hours	364	72.8%
	More than 30 hours	30	6%
Education level	Diploma	36	7.2%
	Bachelor's degree	439	87.8%
	Master's degree	21	4.2%
	Doctorate (PhD) degree	4	0.8%

### Awareness and knowledge about VR/AR in physiotherapy and rehabilitation fields

As presented in Table 2, There were significant differences between final-year physiotherapy students and practicing PTs in their awareness and knowledge of virtual and augmented reality (VR/AR) technologies in physiotherapy and rehabilitation fields; According to the questionnaire, Awareness of VR/AR technology was higher among practicing physiotherapists (62.3%) than students (52.6%) (*p* = 0.017). Attendance at workshops, seminars, or training sessions on VR/AR was also significantly greater among practicing PTs (27.3%) compared to students (12.6%) (*p* = 0.000). When it came to actual use of VR/AR technology during work or training, 18% of practicing PTs reported experience, compared to only 10% of students (*p* = 0.013).While in the responses about sources of knowledge, students most frequently answered internet sources (51.4%) and lectures (45.7%),Workshops were the main source for practicing PTs (66.7%) comparing to 17.1% of students (*p* = 0.000). A significant difference in responses was observed between practicing PTs and students in terms of how frequently they used AR/VR during their work or training. 66.7% of practicing physiotherapists used AR/VR at least once during their work or training, compared to 60% of students used AR/VR several times (*p* = 0.000).

**Table 2:** Comparison of questionnaire responses between students and physiotherapists on awareness and knowledge of VR applications

Questions	Response Distribution:	Final-year students (*n* = 350) n(%)	Practicing PTs (*n* = 150) *n*(%))	Total (*n* = 500) *n*(%))	*P*-value*
Q1.Were you aware of the use of virtual/augmented reality technology in physiotherapy and rehabilitation before this questionnaire?	No/Yes	166 (47.4%)/184 (52.6%)	56 (37.7%)/94 (62.3%)	222(44.4%)/278(55.6%)	0.017
Q2.Have you ever attended a workshop, seminar, or training on the use of VR/AR in physical therapy?	No/Yes	306 (87.4.0%)/44 (12.6%)	109 (72.7%)/41(27.3%)	415 (83.0%)/85(17.0%)	0.000
Q3.Have you ever used VR technology during your work or training in physiotherapy?	No/Yes	315 (90.0%)/35 (10%)	123 (82%)/27(18%)	438 (87.6%)/62(12.4%)	0.013

Questions	Response Distribution:	Final-year students (Total = 35) *n*(%)	Practicing PTs (Total = 27) *n*(%))	Total (Total = 62) *n*(%))	*P*-value*
Q4: If you answered ‘Yes’ to question 3, what are your sources of knowledge about VR/AR reality?	Lectures	16 (45.7%)	6 (22.2%)	22 (35.5%)	0.000
	Workshops	6 (17.1%)	18 (66.7%)	24 (38.7%)	
	Internet Sources	18 (51.4%)	3 (11.1%)	21 (33.9%)	
	Training Sites	2 (5.7%)	0 (0%)	2 (3.2%)	
	Training + Internet	1 (2.9%)	0 (0%)	1 (1.6%)	
Q5.If you answered ‘Yes’ to question 3, how frequently did you use this technology during your work or training?	Once	6 (17.1%)	18 (66.7%)	24 (38.7%)	0.000
	Twice	6 (17.1%)	6 (22.2%)	12 (19.4%)	
	Three Times	1 (2.9%)	1 (3.7%)	2 (3.2%)	
	Several Times	21 (60.0%)	2 (7.4%)	23 (37.1%)	
	Regularly	1 (2.9%)	0 (0%)	1 (1.6%)	

*VR *Virtual Reality, *AR *Augmented Reality, *Practicing PT *Practicing physiotherapists, *n *number of participants, % percentage

*Chi-Square Test; *p*: ≤0,05

### Knowledge of VR/AR applications in the fields of physiotherapy and rehabilitation

As reported in Table 3, For the definition of “technological physiotherapy and rehabilitation,” both groups (57.2%) most frequently identified modern motion tracking, with no significant difference between them (*p* = 0.678).Regarding the primary advantage of using VR/AR and smart technology, the most common answers were the ability to simulate real-life scenarios and the improvement of clinical practice by percentages of 31.8% and 27.4%, respectively and there is no significant difference between students and Practicing PTs (*p* = 0.625).While for disadvantages, high cost was the most frequently reported concern in both groups with 53.0% and this difference was not statistically significant (*p* = 0.980). Both students and Practicing PTs considered neurological (32.8%) and musculoskeletal rehabilitation (30.6%) to be the area’s most likely to use VR/AR applications, with no significant difference observed (*p* = 0.837). Finally, nearly all participants in both groups believed that VR/AR could improve patients’ treatment experience (93.8%, *p* = 0.903).

**Table 3:** Comparison of questionnaire responses between students and physiotherapists on knowledge of VR applications in physiotherapy

Questions	Response Distribution:	Final-year students (*n* = 350) n(%)	Practicing PTs (*n* = 150) *n*(%))	Total ( *n* = 500) *n*(%))	*P*-value*
Q6.Which of the following do you think is included in the term ‘technological physiotherapy and rehabilitation’?	Modern motion tracking (e.g., gait analysis)	196 (56.0%)	90 (60.0%)	286 (57.2%)	0.678
	Virtual reality	44 (12.6%)	19 (12.7%)	63 (12.6%)	
	Artificial intelligence	9 (2.6%)	2 (1.3%)	11 (2.2%)	
	Robotic-assisted rehabilitation	30 (8.6%)	6 (4.0%)	36 (7.2%)	
	Assistive emergency devices	4 (1.1%)	1 (0.7%)	5 (1.0%)	
	Smart prosthetics	53 (15.1%)	24 (16.0%)	77 (15.4%)	
	Advanced biosensors	8 (2.3%)	5 (3.3%)	13 (2.6%)	
	All of the above	6 (1.7%)	3 (2.0%)	9 (1.8%)	
Q7.In your opinion, what is the most important advantage of using VR/AR tools and smart technology in the fields of physiotherapy and rehabilitation?	Improving students’ clinical practice	97 (27.7%)	40 (26.7%)	137 (27.4%)	0.625
	Saving time in patient evaluation	26 (7.4%)	15 (10.0%)	41 (8.2%)	
	Reducing treatment costs	9 (2.6%)	1 (0.7%)	10 (2.0%)	
	Ability to simulate real-life scenarios	109 (31.1%)	50 (33.3%)	159 (31.8%)	
	Ability to provide digital data	3 (0.9%)	2 (1.3%)	5 (1.0%)	
	Increasing patient engagement	22 (6.3%)	12 (8.0%)	34 (6.8%)	
	Improving therapeutic outcomes	84 (24.0%)	30 (20.0%)	114 (22.8%)	
Q8.In your opinion, what is the main disadvantage of using VR/AR tools in the fields of physiotherapy and rehabilitation?	High cost	183 (52.3%)	82 (54.7%)	265 (53.0%)	0.980
	Need for technical support/maintenance	55 (15.7%)	20 (13.3%)	75 (15.0%)	
	Requires equipment and trainings	45 (12.9%)	19 (12.7%)	64 (12.8%)	
	Space requirements	9 (2.6%)	3 (2.0%)	12 (2.4%)	
	Requires expertise in the usage	48 (13.7%)	22 (14.7%)	70 (14.0%)	
	Reduces job opportunities	1 (0.3%)	1 (0.7%)	2 (0.4%)	
	Reduces human interaction	1 (0.3%)	0 (0%)	1 (0.2%)	
	All of the above	8 (2.3%)	3 (2.0%)	11 (2.2%)	
Q9.Which area of physiotherapy and rehabilitation do you think VR/AR technology most suitable for or commonly used in?	Neurological rehabilitation	113 (32.3%)	51 (34.0%)	164 (32.8%)	0.837
	Musculoskeletal rehabilitation	107 (30.6%)	46 (30.7%)	153 (30.6%)	
	Sports injury rehabilitation	53 (15.1%)	24 (16.0%)	77 (15.4%)	
	Pediatric rehabilitation	7 (2.0%)	4 (2.7%)	11 (2.2%)	
	Cardiopulmonary rehabilitation	43 (12.3%)	16 (10.7%)	59 (11.8%)	
	Geriatric rehabilitation	8 (2.3%)	5 (3.3%)	13 (2.6%)	
	Pelvic health rehabilitation	19 (5.4%)	4 (2.7%)	23 (4.6%)	
Q10.Do you believe that using VR/AR can improves patients’ experience in therapy?	No/Yes:	22(6.3%)/328(93.7%)	9(6%)/141(94%)	31(6.2%)/469(93.8)	0.903

*VR* Virtual Reality, *AR* Augmented Reality, *Practicing PTs* Practicing physiotherapists, *n * number of participants, % percentage

*: Chi-Square Test, *p*: ≤0,05

### Future directions and training needs in virtual reality for physiotherapy and rehabilitation

Overall, nearly all participants in both groups اhighly expressed neutral opinions regarding the ethical aspects of using of VR in physiotherapy with percentage of 51.2% (*p* = 0.549). A substantial proportion of respondents of both groups believe that current educational and training programs do not effectively educate them for employing these technologies in clinical practice and needs for improvements (52.4%, *p* = 0.323).

Responses to the question of the potential impact of VR/AR technology on future employment opportunities for physiotherapists. Specifically, 10.0% of respondents strongly disagreed and 13.2% disagreed that VR/AR would affect employment prospects, while 36.4% remained neutral. On the other hand, 17.2% agreed and 23.2% strongly agreed that VR/AR could influence future job opportunities in the physiotherapy field. with no significant difference between the two groups (*p* = 0.514). 89% of students and practicing PTs prefer a combination of traditional and VR methods (*p* = 0.971) when asked about their preferred method of practice.

There was significant interest in additional training on VR applications in physiotherapy among both groups by percentage of 76% expressed agreement or strong agreement regarding the necessity for further training (*p* = 0.328). The utilization of VR in a variety of rehabilitation fields was a top preference for training materials, followed by patient engagement and motivation, and progress monitoring & analysis in both groups; at 37.8%, 17.4% and 14.4%, respectively.Additionally, with no statistically significant differences were found between students and practicing PTs across any of this sections items (all p-values > 0.05), as presented on Table 4 below.

**Table 4:** Comparison of responses on future directions and training needs in VR/AR for physiotherapy

Questions	Response Distribution:	Final-year students (*n* = 350) n(%)	Practicing PTs (*n* = 150) *n*(%))	Total (*n* = 500) *n*(%))	*P*-value
Q11.What is your opinion on the ethical aspects of using VR/AR in physiotherapy?	Positive	160 (45.7%)	74 (49.3%)	234 (46.8%)	0.549*
	Neutral	184 (52.6%)	72 (48.0%)	256 (51.2%)	
	Negative	6 (1.7%)	4 (2.7%)	10 (2.0%)	
Q12.Do you believe that current education and training programs adequately prepare you to use these technologies?	No	131 (37.4%)	66 (44.0%)	197 (39.4%)	0.323*
	Needs Improvement	191 (54.6%)	71 (47.3%)	262 (52.4%)	
	Yes	28 (8.0%)	13 (8.7%)	41 (8.2%)	
Q13.Do you think future employment opportunities for physiotherapists and rehabilitation specialists will decrease with the use of VR/AR?	Strongly Disagree *n*(%)	36 (10.3%)	14 (9.3%)	50 (10.0%)	0.514**
	Disagree *n*(%)	52 (14.9%)	14 (9.3%)	66 (13.2%)	
	Neutral *n*(%)	126 (36.0%)	56 (37.3%)	182 (36.4%)	
	Agree *n*(%)	64 (18.3%)	22 (14.7%)	86 (17.2%)	
	Strongly Agree *n*(%)	72 (20.5%)	44 (29.3%)	21 (23.2%)	
Q14.If you had the opportunity to perform both of the following practices, please indicate which one you would prefer to do?	Only VR/AR techniques	23 (6.57%)	9 (6.0%)	32 (6.4%)	0.971*
	Only Traditional PT techniques	16 (4.57%)	7 (4.7%)	23 (4.6%)	
	Both (traditional + VR/AR technology)	311 (88.85%)	134 (89.3%)	445 (89.0%)	
Q15:Would you like to receive more training on the use of VR/AR in physiotherapy?	Strongly Disagree *n*(%)	6 (1.7%)	3 (2.0%)	9 (1.8%)	0.328**
	Disagree *n*(%)	8 (2.3%)	21 (14%)	29 (5.8%)	
	Neutral *n*(%)	58 (16.6%)	22 (14.7%)	80 (16.0%)	
	Agree *n*(%)	160 (45.7%)	66 (44.0%)	226 (45.2%)	
	Strongly Agree *n*(%)	118 (33.7%)	38 (25.3%)	156 (31.2%)	
Q16.In which area would you like to receive additional training on smart technology?	VR/AR in various rehab fields	128 (36.6%)	61 (40.7%)	189 (37.8%)	0.534*
	VR/AR in Patient motivation & engagement	56 (16.0%)	31 (20.7%)	87(17.4%)	
	VR/AR in Progress monitoring & analysis	49 (14.0%)	23 (15.3%)	72(14.4%)	
	VR/AR in Patient assessment	40 (11.4%)	12 (8.0%)	52(10.4%)	
	Specialized program development in VR/AR	35 (10.0%)	11 (7.3%)	46(9.2%)	
	VR/AR in Remote patient rehab	19 (5.4%)	7 (4.7%)	26(5.2%)	
	VR in for team communication	17 (4.9%)	4 (2.7%)	21(4.2%)	
	All of the above	6 (1.7%)	1 (0.7%)	7(1.4%)	

*VR* Virtual Reality, *AR* Augmented Reality, *Practicing PTs* Practicing physiotherapists, *n* number of participants, % percentage

*: Chi-Square Test, *p* ≤0,05, **: Mann-Whitney U; *p* ≤0,05

### Participants’ opinions on the future of VR/AR technologies

To determine the anticipated future role of VR/AR in physiotherapy and rehabilitation, as well as the Participants’ Opinions on their future integration as reported on Table 5.Most final-year students and Practicing PTs (51.%) agreed with the statement that virtual reality (VR) will become the ideal tool or technique in physiotherapy and rehabilitation (Q17). There was no statistically significant difference between the two groups (*p* = 0.358).Responses to the question about the future significance of virtual reality in physiotherapy and rehabilitation (Q18) showed a similar response, with 57% of students and Practicing PTs agreeing and there isnot a significant difference between them (*p* = 0. 0.838). 56.7% of students and Practicing PTs agreeing that VR/AR will improve efficiency in the fields of physiotherapy and rehabilitation (Q19). There was no statistically significant difference between the groups (*p* = 0.612). Additionally, 91.8% of students and Practicing PTs indicated there is a need to develop specialized educational programs focused on the use of modern technology in physiotherapy (*p* = 0.338) in Q20.

**Table 5:** Comparison of student and physiotherapist opinions on the future of VR/AR in physiotherapy

Question	Group	Strongly Disagree *n*(%)	Disagree *n*(%)	Neutral *n*(%)	Agree *n*(%)	Strongly Agree *n*(%)	*P* value
Q17.Do you think VR/SR will become an ideal tool or technique in the fields of physiotherapy and rehabilitation in the future?	Final-year students (*n* = 350) *n*(%)	12 (3.4%)	14 (4.0%)	115 (32.9%)	174 (49.7%)	35 (10.0%)	0.358**
	Practicing PTs (*n* = 150) *n*(%)	6 (4.0%)	4 (2.7%)	43 (28.7%)	81 (54.0%)	16 (10.7%)	
	Total (*n* = 500) *n*(%)	18 (3.6%)	18 (3.6%)	158 (31.6%)	255 (51.0%)	51 (10.2%)	
Q18.Do you think that VR/AR will be an important addition to the fields of physiotherapy and rehabilitation in the future?	Final-year students (*n* = 350) *n*(%)	5 (1.4%)	18 (5.1%)	82 (23.4%)	197 (56.3%)	48 (13.7%)	0.838**
	Practicing PTs (*n* = 150) *n*(%)	4 (2.7%)	7 (4.7%)	31 (20.7%)	88 (58.7%)	20 (13.3%)	
	Total (*n* = 500) *n*(%)	11 (2.2%)	25 (5.0%)	113 (22.6%)	285 (57.0%)	66(13.2%)	
Q19.Do you think that VR/AR will improve efficiency in the fields of physiotherapy and rehabilitation?	Final-year students (*n* = 350) *n*(%)	5 (1.4%)	12 (3.4%)	76 (21.7%)	196 (56.0%)	61 (17.4%)	0.602**
	Practicing PTs (*n* = 150) *n*(%)	2 (1.3%)	4 (2.7%)	30 (20.0%)	87 (58.0%)	27 (18.0%)	
	Total (*n* = 500) *n*(%)	7(1.4%)	16(3.2%)	106(21.2%)	283(56.6%)	88(17.6%)	

Question	Group	Response Distribution	*n*(%)		*n*(%)		*P*-value
Q20.Do you think there is a need to develop specialized educational programs focused on the use of modern technology in physiotherapy?	Final-year students)*n* = 350) *n*(%)	No/yes	28 (8.0%)		322 (92.0%)		0.803*
	Practicing PTs (*n* = 150) *n*(%)	No/yes	13 (8.7%)		137 (91.3%)		
	Total (*n* = 500) *n*(%)	No/yes	41(8.2%)		459 (91.8%)		

*VR *Virtual Reality, *AR* Augmented Reality, *Practicing PTs* Practicing physiotherapists, *n* number of participants, % percentage

*:Chi-Square Test, *p*: ≤0,05, **: Mann-Whitney U, *p*: ≤0,05

## Discussion

This study explored and compared the awareness and perceptions of VR/AR technologies in physiotherapy among final-year students and practicing clinicians in Palestine. The findings indicate a moderate overall level of awareness, with clinicians reporting greater familiarity than students. While these results align with international observations of gradual VR adoption in rehabilitation, they also highlight unique challenges in the Palestinian context, where systemic, economic, and infrastructural barriers restrict integration into routine practice. These findings contribute to the literature on immersive technology adoption, particularly in low-resource settings where infrastructure and training limitations impede implementation [8, 9].

### Awareness and exposure to VR/AR technologies

The results revealed that awareness of VR/AR in physiotherapy was significantly higher among practicing physiotherapists compared to students, suggesting that clinical experience may play a role in increasing familiarity with emerging technologies. This finding aligns with previous research which found that clinicians with more years of practice were more likely to engage with novel rehabilitation technologies due to greater exposure to professional development opportunities [6]. Our study further supports this notion, as practicing clinicians reported higher attendance in VR/AR-related workshops and seminars, reinforcing the importance of workplace-based training in bridging the knowledge gap.

Despite this, actual use of VR/AR tools remained low in both groups (18% among clinicians vs. 10% among students), mirroring global trends where adoption lags behind awareness. For instance, a survey of German physiotherapists found that while over 60% were aware of therapeutic VR, only 2.7% had used it in the past year underscoring systemic barriers such as cost, limited accessibility, and insufficient training [7]. Similarly, a UK study of pediatric physiotherapists reported that 93% had never implemented VR in practice, with low usage attributed to factors like lack of time, resources, and institutional support [10].

In Palestine, however, the barriers may be amplified by limited institutional support, scarcity of specialized training, and constrained healthcare budgets. The reliance of students on internet resources, contrasted with clinicians’ greater access to workshops, reflects the lack of structured curricular integration in academic programs. This gap underscores the need for embedding VR/AR competencies into physiotherapy education, ensuring early and equitable exposure across student cohorts. Cost and technical support emerged as dominant barriers, which is unsurprising given Palestine’s constrained resources. However, recent studies have highlighted promising low-cost or open-source VR systems designed for rehabilitation that may offer feasible alternatives in such contexts. For example, smartphone-based VR platforms or motion-tracking devices like Kinect have demonstrated effectiveness in stroke and musculoskeletal rehabilitation at a fraction of the cost of high-end systems [11]. Exploring the applicability of these scalable, affordable solutions in Palestine could provide a practical pathway toward adoption.

Study findings also reveal differences in sources of VR/AR knowledge: students relied heavily on internet resources and lectures, whereas clinicians primarily cited workshops. This disparity underscores the need for academic curricula to integrate hands-on VR/AR training, supporting the argument of Laver et al. (2017), who emphasized that early exposure during education fosters long-term competency in rehabilitation technology [12].

### Understanding and perceptions of VR/AR applications

Despite differences in exposure, both students and clinicians demonstrated a similar conceptual understanding of VR/AR applications, particularly in motion tracking and simulation-based rehabilitation. These perceptions align with global studies, which highlight the efficacy of VR in enhancing motor learning, engagement, and therapeutic outcomes in neurological and musculoskeletal rehabilitation [13]. Notably, our participants identified stroke rehabilitation and pain management as key areas for VR/AR application, which consistent with a meta-analysis that reported significant improvements in upper limb function among stroke patients using VR therapies [14].

However, cost emerged as the primary barrier (reported by >50% of participants), consistent with research from low- and middle-income countries [8]. Additionally, concerns about technical support and training needs reflect broader challenges identified in the literature, including the necessity for institutional infrastructure and workforce upskilling [9]. These barriers may be particularly pronounced in Palestine, where healthcare resources are often limited, reinforcing the need for cost-effective VR/AR solutions and scalable training programs.

### Future directions and training needs

A strong consensus emerged regarding the inadequacy of current education in preparing students and clinicians for VR/AR integration. Over half of respondents called for curricular improvements, echoing recommendations by Ødegaard et al. (2021), who argued that physiotherapy programs must evolve to include digital competencies including immersive technologies like VR as fundamental components of professional training [15]. Interestingly, while ethical concerns were not a dominant issue in our study, the rapid advancement of VR/AR particularly with AI integration and remote patient monitoring warrants further ethical scrutiny [16].

The preference for blended practice models (89% favoring a mix of traditional and VR-based therapy) aligns with the findings of Krasovsky et al. (2020), who reported that clinicians view VR as a supplementary rather than a replacement tool [17]. Moreover, the overwhelming interest in additional training reflects a readiness to adopt VR/AR, provided that structured upskilling opportunities are available.

### Limitations and methodological considerations

Several methodological limitations should be acknowledged. First, the use of convenience sampling may have introduced selection bias, as participants more interested in technology may have been more likely to respond. Second, the cross-sectional design precludes conclusions about causality or temporal trends, which are especially relevant in a rapidly evolving field such as digital rehabilitation. Third, reliance on self-reported data raises the risk of over- or under-estimating actual competency and usage, given the absence of objective assessments. Future research should address these limitations through longitudinal designs, probabilistic sampling, and performance-based measures of VR competency.

### Contribution and regional significance

Despite these limitations, this study makes a notable contribution as the first investigation of VR/AR awareness and perceptions among physiotherapy students and clinicians in Palestine. Its novelty lies in filling a significant knowledge gap in a low-resource, conflict-affected setting, where research on digital health adoption is scarce. By situating Palestinian findings within international comparisons, the study extends debates on VR adoption to regions often underrepresented in rehabilitation technology research. This perspective is essential for shaping global strategies that are inclusive of diverse socioeconomic and political realities.

## Conclusion

Awareness of VR/AR in physiotherapy is gradually emerging in Palestine; however, widespread adoption remains limited due to cost, infrastructure challenges, and insufficient training and institutional support. Clinicians currently demonstrate greater familiarity than students, highlighting the need for earlier curricular integration of digital competencies. As digital rehabilitation continues to gain global prominence, proactive investment in training, affordable technologies, and public–private collaboration will be essential to ensure equitable access. Future research should evaluate the impact of VR/AR training interventions and explore contextually adapted, cost-effective strategies and policy measures to sustainably integrate immersive technologies into Palestinian rehabilitation services.

## Data Availability

The datasets generated and/or analyzed during the current study are not publicly available due to participant confidentiality but are available from the corresponding author on reasonable request.

## Abbreviations

VR: Virtual reality
AR: Augmented reality
PTs: Practicing physiotherapists
IRB: Institutional Review Board
N: Number of participants
%: Percentage
